# Parameter design and experiment of rotary plate pineapple fruit picker

**DOI:** 10.3389/fpls.2025.1575648

**Published:** 2025-05-05

**Authors:** Jiangyi Qiu, Jieli Duan, Zhaoxin Zhang, Zhong Xue

**Affiliations:** ^1^ College of Engineering, South China Agricultural University, Guangzhou, China; ^2^ South Subtropical Crops Research Institute, Chinese Academy of Tropical Agricultural Sciences, Zhanjiang, China; ^3^ College of Engineering and Technology, Shanxi Agricultural University, Jinzhong, China

**Keywords:** harvest, design, emulation, pineapple, fruit-picker

## Abstract

Research on pineapple harvesting machinery remains in its early stages, and the design and development of such machinery play a crucial role in advancing mechanization and automation within the pineapple industry. This study designs the rotary plate pineapple fruit harvester’s cutting table structure based on the physical and mechanical properties of pineapple plants. The design process includes simulating manual harvesting techniques. Initially, a thorough analysis was performed to determine the structural parameters of the cutting table’s key components, including the plucking speed ratio, the height of the fruit-picking plate from the ground, and the forward speed of the harvester. A stress analysis of the stem during harvesting was then conducted based on the principle of large deflection. This analysis allowed for the examination of two conditions that occur during fruit picking: (1) the stress state when the fruit and stem are separated at the end of the stem connection, and (2) the stress state when the stem breaks at a point along its length, leading to separation. A simplified rigid-flexible coupling model of the cutting platform and pineapple plant was developed using simulation software, followed by a three-factor, five-level virtual response surface analysis. This analysis was conducted to determine the optimal parameter combinations that would ensure the effective force exerted by the picking plate on the pineapple exceeds the average separation force of 60.45 N, while minimizing the peak force. The optimal parameters included a pulling ratio of 1.8, a picking plate center height of 890 mm above the ground, and a forward speed of the harvester of 0.4 m/s. Subsequent field experiments were carried out to validate the effectiveness of the optimal picking plate parameters. The field experiments with the optimal parameter combination demonstrated that the pineapple fruit picking rate was 71.3%, and the damage rate was 14.8%. The study’s results provide valuable insights for future research on pineapple harvesting machinery.

## 1 Introduction

Pineapple is one of the four principal tropical fruits, alongside banana, mango, and coconut. It is the most economically significant plant in the Bromeliaceae family and ranks as the second-largest economic tropical fruit globally (Li et al., 2022; Sapak and Nusaibah, 2024). China is both one of the top ten leading pineapple producers and a major consumer. China’s pineapple cultivation spans approximately 67,000 hectares, yielding an annual output exceeding 1.8 million tons (Zhong et al., 2021; Villalobos et al., 2023). Given the unique value of pineapple, its market is still evolving, and the scale of its cultivation continues to expand. However, at present, pineapple planting, management, and harvesting rely entirely on manual labor, and the associated labor costs account for 40% of the total pineapple production cost (Xiangfeng et al., 2019; He et al., 2024). Furthermore, the pineapple corolla and the leaf edges are covered with numerous thorns. Additionally, the harvest is highly seasonal, with the peak harvesting period typically lasting only two weeks, which further exacerbates the challenges associated with pineapple harvesting. Currently, a significant number of young, capable laborers in rural areas are losing employment, and labor costs are steadily rising. These issues have significantly hindered the further development of the pineapple industry, while the demand for mechanized harvesting equipment in China has become increasingly urgent (Brainy et al., 2023; Meng et al., 2023; Zhang et al., 2024).

Current domestic and international research on pineapple harvesting machinery and equipment spans a wide range of technical aspects. Based on their level of automation, these systems can be classified into semi-automatic, fully automated, and intelligent harvesting equipment. Among the semi-automatic systems, further categorization includes pineapple transfer equipment and manually assisted harvesting equipment (He et al., 2024). Currently, foreign fruit farms predominantly utilize large-scale transfer platforms, supplemented by manual pineapple picking methods. For instance, in Mexico, pineapples are harvested through a transport arm connected to a belt conveyor system, while in Costa Rica, pineapples are picked via an extending arm with rotating fingers that clamp the pineapple crown buds, followed by centralized transmission (Paull et al., 2017; Zhong et al., 2021). Most manually assisted harvesting devices employ fruit clamping combined with cutting and breaking mechanisms to harvest the fruits (Zhang et al., 2018; Ma et al., 2020; Qing, 2020; Bhat and Chayalakshmi, 2021; Kahandage et al., 2021; Mazlan et al., 2024). Indian researchers, Kahandage et al., have developed a hand-held assisted picking device capable of securely clamping pineapples of varying shapes and sizes. The device operates by allowing the user to clamp and cut pineapples through a lever mechanism located at the operating end of the handle. The combination of the lever and clamping claw ensures secure clamping of pineapples of all shapes and sizes during the cutting process. The device achieves a harvesting capacity of 385 fruits per hour, with a harvesting efficiency of 84% (Kahandage et al., 2021). In addition, fruit clamping and stem cutting can be controlled by a microcontroller to accomplish the harvesting of pineapple fruits of different sizes and shapes, reducing the burden of operators (Bhat and Chayalakshmi, 2021). Qing Zhong designed a flexible assisted picking device that adapts to the size and shape of pineapples. It incorporates a triangular-shaped sharp cutter, optimized for cutting pineapple stems, and The gravity locking device is also designed to use gravity to clamp the pineapple after picking. This design is particularly beneficial for domestic pineapple harvesting (Qing, 2020). However, manually assisted harvesting equipment requires operators to possess a high level of proficiency to achieve optimal harvest and minimize damage rates. Additionally, most manually assisted equipment lacks a gravity compensation mechanism, which can lead to operator fatigue during use (Yichen et al., 2018). Pineapple automatic harvesting equipment primarily utilizes mechanized systems to efficiently pick and transport fruits. The key function of this equipment is to automate the harvesting process by incorporating an automatic harvesting mechanism at the front end and using a transportation system to collect the harvested fruits. Guo and colleagues developed a robotic arm-based automatic harvesting system featuring remote operator control and two three-degree-of-freedom arms for automatic identification and picking. The device employs a scoop-shaped clamping mechanism, where the packaging clamping space exceeds the size of the fruit, thereby preventing damage during clamping. Additionally, the overall gripping action is more stable (Guo et al., 2021). Liu et al. investigated a harvesting machine that eliminates the need for fruit clamping, leveraging the characteristics of the shedding zone between the ripe fruit and the stalk. This approach mimics manual harvesting by generating an appropriate breaking torque through the relative motion between the flexible finger roller and the pineapple, which effectively separates the pineapple from the shedding layer (Liu et al., 2022). Pineapple intelligent picking equipment primarily includes fruit-picking robots and harvesting devices that integrate intelligent systems, based on conveyorized or automatic picking equipment (He et al., 2024). The fruit-picking robot consists mainly of a vision system, a robotic arm, an end-effector, a travelling system, and a control system. However, pineapple fruit-picking robots vary in vision systems, robotic arm structures, and end-effector mechanisms, leading to differences in operational performance. Vietnamese scholars, including Nguyen Pham Thuc Anh et al., developed a pineapple picking device featuring a Cartesian manipulator with three degrees of freedom, supplemented by machine vision recognition. The robot achieved a recognition accuracy of 90.82%, and by optimizing the dynamics and motion control of the manipulator and end-effector, the picking success rate improved to 95.56%, with each pineapple being picked in approximately 12 seconds (Anh et al., 2020; Hoang et al., 2022). Du et al. utilized a sensor-controlled manipulator clamping structure to secure the fruit and prevent stem deflection, gripping method using movement from the crown bud to the center of the fruit, thereby avoiding stem damage or accidental injury to the fruit during the cutting process by the disc saw. The end-effector exhibited an average picking time of 14.76 seconds, with a fruit loss rate of 5%, a plant damage rate of 0%, and a fruit drop rate of 1.7% (Du et al., 2019). However, research on pineapple intelligent picking technology is still in its early stages, with limited advancements in technology integration systems. Issues such as poor recognition and positioning accuracy, low picking success rates, and suboptimal picking efficiency must be addressed. Further research is required to refine fruit recognition and positioning methods, as well as to optimize the structural design of the end-effector and the performance of fruit separation techniques (He et al., 2024).

However, research on pineapple harvesting equipment, both domestically and internationally, remains in the early and exploratory stages. Numerous challenges persist regarding the use, harvesting efficiency, and promotion of the currently developed machines. Considerable progress is still required before the mechanization of pineapple picking can be fully realized. Therefore, further exploration and research on pineapple picking machines is warranted. This study builds upon existing theoretical analyses and research, considering the geometry of the pineapple fruit, calyx union, and stem fragility, as well as other biological characteristics. Utilizing the principles of bionics and agricultural machinery design theory, this study aims to analyze and design the structural parameters of the cutter platform picking plate. Additionally, based on the principle of large deflection, the study calculates the stem bending stress deformation and utilizes Adams simulation to determine the optimal rotational speed of the cutting table and walking speed. The results are validated through field experiments. The development of this machine will revolutionize the traditional pineapple picking method and significantly reduce the high labor intensity associated with pineapple harvesting.

## 2 Self-propelled pineapple harvester with working process

### 2.1 Self-propelled pineapple harvester basic structure

**Figure 1:**
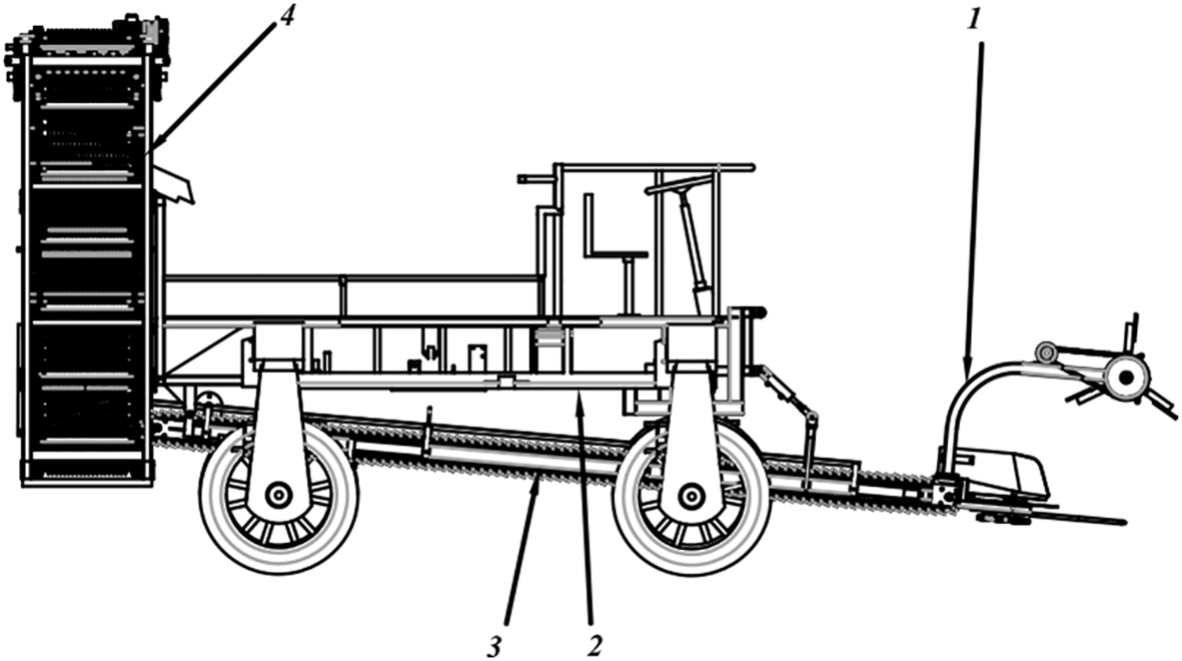
Structure of the pineapple harvester. 1. Cutting table 2. Car body 3. Transportation structure 4. Collection structure.

The self-propelled pineapple harvester primarily consists of a cutting platform, a transportation system, a collection structure, and a traveling mechanism, as illustrated in **Figure 1**. The cutting platform and the transportation structure are linked by a telescopic plate, which can be adjusted to vary the fore-and-aft distance between the picking mechanism and the transportation system. The transportation and locomotion mechanism is connected to a hydraulic pump, which adjusts the height of the cutting platform relative to the ground based on the varying heights of different plant varieties. The entire system is powered by a diesel engine, with the disc saw, transportation mechanism, and collection system driven by a hydraulic motor, while the fruit-picking mechanism is powered by a DC motor.

### 2.2 Harvester work process

Prior to operation, the harvester adjusts the telescopic plate and hydraulic pump to ensure that the cutting platform is set at the correct height and maintains an appropriate distance from the transportation mechanism, both front and rear. The traveling speed is then adjusted to the desired value, and the harvester proceeds along the ridge to begin operation. During the picking process, the fruit-picking plate rotates at a certain angle and makes contact with the fruit. At this moment, the fruit-picking plate moves with a backward speed, applying a backward force on the fruit, which, in turn, causes the stem to bend and deform, generating stress. When the stress on the stem reaches its threshold, the fruit detaches from the stem, completing the picking process. Following the picking process, the fruit moves backward under the influence of the picking plate and falls onto the transportation structure. Once transported to the end of the transportation structure, the fruit falls into the collection mechanism and is gathered into a collection box by the collection structure. To minimize damage, the parts of the harvester that come into contact with the fruit are fitted with flexible materials.

## 3 Structural design of the cutting table

**Figure 2:**
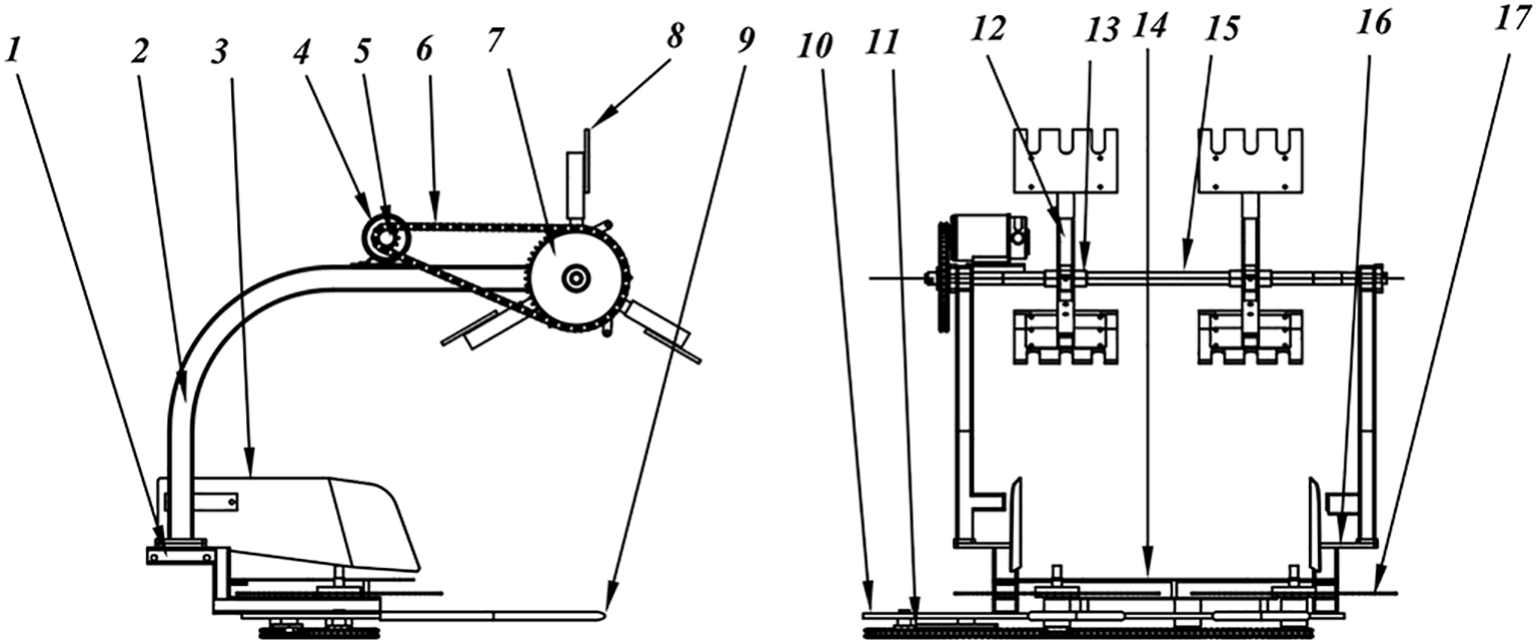
Structure of the cutting table. 1. lower bracket 2. upper bracket 3. stopper 4. motor 5. active sprocket of the picking plate 6. chain of the picking plate 7. slave sprocket of the picking plate 8 picking plate 9. v-bar 10. large pad 11. chain of the circular saw 12. connecting arm 13. fixed part of the connecting arm 14. lower gear plate 15. rotating shaft 16. connecting plate 17. circular saw.

**Figure 2** illustrates the structure of the cutting table. The fruit-picking plate is the primary working component of the cutting table mechanism, designed to apply a backward force to pineapple plants that are either growing normally or slightly tilting, thereby simulating the manual method of pineapple harvesting. To minimize damage to the pineapple, the part of the fruit picking plate that contacts the fruit is made of rubber.

### 3.1 Picking mechanism key parameters and motion analysis

The key parameters of the picking mechanism primarily consist of the plucking speed ratio, the height of the center of the picking plate from the ground, the forward displacement of the center of the picking plate, the rotational speed of the picking plate, and the rotational radius of the picking plate. The initial step involves analyzing the motion of the fruit picking plate during the picking process, with the aim of evaluating and optimizing the parameters of the picking mechanism.

#### 3.1.1 Kinematic analysis of a fruit picking board

**Figure 3:**
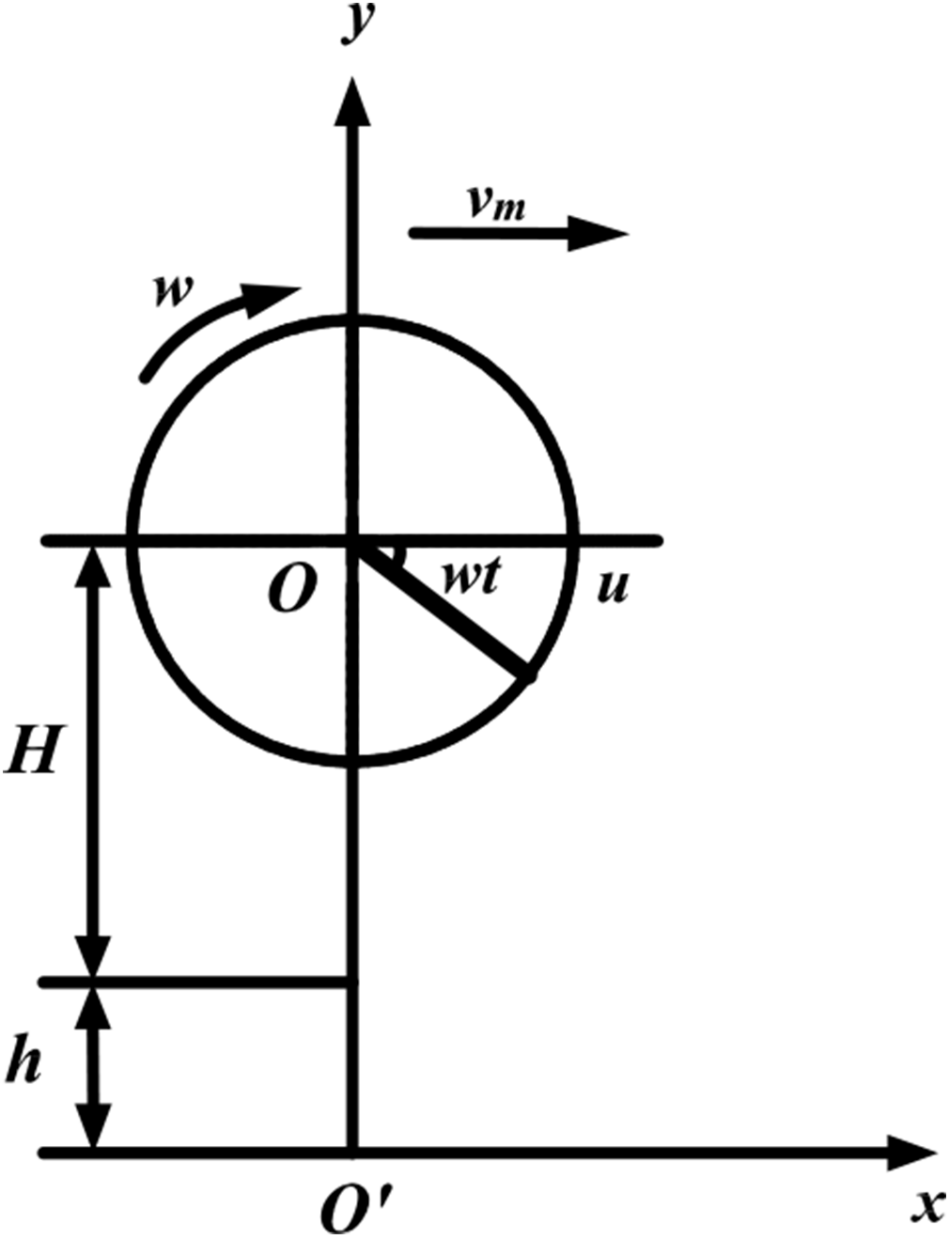
Schematic diagram of the movement of the fruit-picking plate.


The motion of the picking plate results from the combined effect of the forward motion of the harvester and the rotational motion of the picking plate relative to the cutting table. To facilitate the analysis of the motion of the fruit-picking plate during harvesting, a simple Cartesian coordinate system is established, and the motion of the plate is decomposed. The projection of the center of rotation of the fruit-picking plate onto the ground serves as the coordinate origin, with the forward direction of the harvester aligned with the x-axis, and the direction perpendicular to the forward direction corresponding to the y-axis, as shown in **Figure 3**. A point located at the outer edge of the fruit picking plate begins to rotate from the horizontal position u. The equation of its trajectory is:

(1) $$\{\begin{array}{c} \text{x}={\text{v}}_{\text{m}}\text{t}+\text{R}\text{cos}\text{ω}\text{t}\\ \text{y}=\text{H}+\text{h}-\text{R}\text{sin}\text{ω}\text{t} \end{array}$$

Here, *x* represents the horizontal coordinate of a point on the outer edge of the fruit-picking plate (in mm), and *y* denotes the vertical coordinate of a point on the same edge (in mm). *r* is the rotational radius of the fruit-picking plate (in mm), and *ω* is the rotational angular velocity (in rad/s). *H* represents the vertical distance from the center of rotation of the fruit-picking plate to the disc saw (in mm), and ℎ denotes the perpendicular distance from the disc saw to the ground (in mm).

The derivation of Equation 1 yields the velocity at a point on the outer edge as follows:


(2) $$\{\begin{array}{c} {\text{v}}_{\text{x}}={\text{v}}_{\text{m}}-\text{R}\text{ω}\text{sin}\text{ω}\text{t}\\ {\text{v}}_{\text{y}}=-\text{R}\text{ω}\text{cos}\text{ω}\text{t} \end{array}$$

In the figure, *v_m_* represents the forward speed of the picker (in m/s), *ω* denotes the rotational angular velocity of the fruit-picking plate (in rad/s); *H* and ℎ refer to the vertical distances from the fruit-picking plate to the disc saw and from the disc saw to the ground, respectively (in mm).

#### 3.1.2 Normal working conditions for fruit picking boards

**Figure 4:**
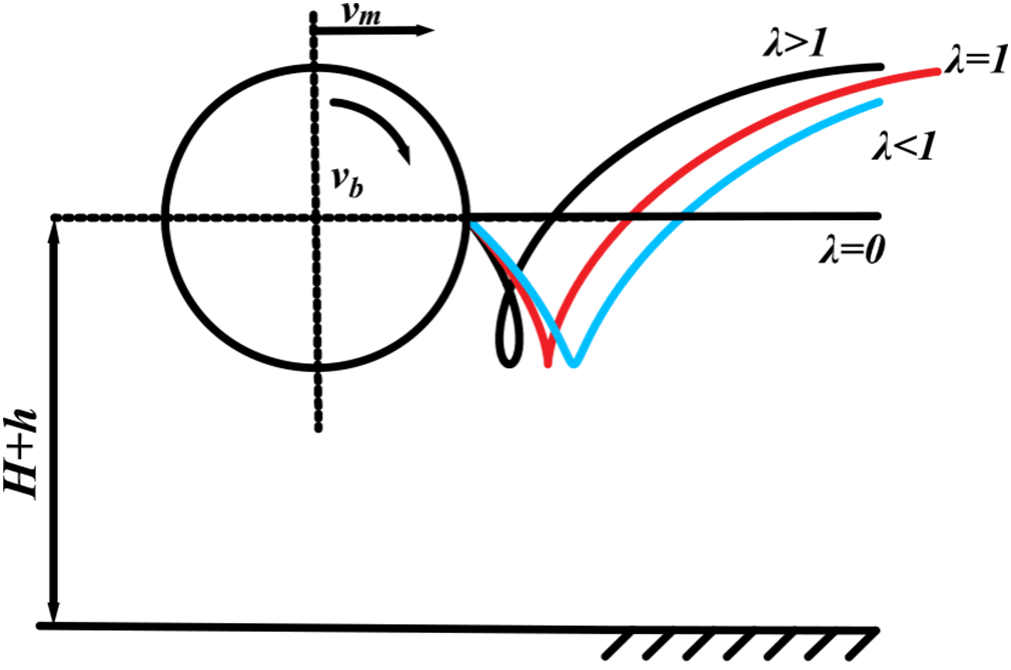
Motion trajectories for different λ.

In fruit picking, the fruit-picking board must have a backward component of velocity in the horizontal direction to facilitate the straightening of the plant, as well as fruit peeling, pushing, and other tasks. The trajectory of the fruit-picking board significantly affects its ability to function properly. The motion trajectories of different *λ* are shown in **Figure 4**. The ratio of the circumferential speed *v_b_* of the fruit picking plate to the forward speed *v_m_* of the harvester defines the plucking speed ratio *λ*, i.e., $\lambda =\frac{v_{b}}{v_{m}}$ . When *λ*≤1, the trajectory of the fruit-picking plate follows a typical pendulum path, does not form a closed snap ring, lacks a horizontal backward component of velocity, and does not exert a peeling or pushing effect on the fruit. When λ>1, the trajectory of the fruit-picking plate follows a residual pendulum path, forming a closed snap ring (Qing et al., 2021). In this case, the fruit picking board exhibits horizontal backward speed, enabling it to peel and push the fruit. Therefore, the necessary condition for regular fruit picking by the fruit-picking plate is *λ*>1.

### 3.2 Parameter design of fruit picking board

This paper presents an analysis and design of the key parameters for the fruit-picking plate, considering the following physical and mechanical properties of pineapple plants and fruits.

#### 3.2.1 Fruit-picking plate plucking speed ratio and diameter

In reference to the design of the paddle wheel of the rice-wheat combine harvester, the fruit-picking plate must fulfill two key requirements for the pineapple picking operation:

(1) The horizontal component of the velocity of the fruit picking plate when it contacts the pineapple should be zero to avoid causing excessive impact on the fruit and prevent pushing it. According to Equation 2, we obtain:


(3) $$\text{sin}\omega t=\frac{v_{m}}{\omega R}=\frac{1}{\lambda }$$

(2) The fruit-picking plates should be positioned at the upper center of gravity of the pineapple plant during the fruit-picking process. Based on available information, it is known that the center of gravity of the pineapple plant is approximately located above the top of the plant (Liu et al., 2023). According to Equation 3, the following holds:


(4) $$\text{R}=\text{R}\text{sin}\text{ω}\text{t}+\frac{\left(\text{L}-\text{h}\right)}{5}=\frac{\text{λ}\left(\text{L}-\text{h}\right)}{5\left(\text{λ}-1\right)}$$

The pitch of the trochoidal buckle between the two adjacent fruit picking plates is


(5) $$\text{S}={\text{v}}_{\text{m}}\frac{2\text{π}}{\text{z}\text{ω}}=\frac{2\text{π}\text{R}}{\text{z}\text{λ}}$$

To ensure that no fruits are missed during the picking process, the pitch should be at least equal to the minimum plant spacing, i.e.,:


(6) $$\text{S}\leq {\text{S}}_{\text{min}}$$

Where *S*_min_ represents the minimum plant spacing, assumed to be 400 mm; *L* is the plant height, assumed to be 778.5 mm; *h* denotes the stubble height, taken to range from 200 to 300 mm; and *z* is the number of fruit-picking plates, assumed to be three in this case.

Combined Equations 4–6, the solution yields *λ*≥1.6. By setting *λ*=1.6, the fruit-picking plate radius *R* is calculated to range from 255.2 to 281.9 mm. Considering variations in plant height and uneven ground, we adopt *R*=280 mm in this case.

#### 3.2.2 Fruit picking board mounting height

In order to ensure that the picking board and the pineapple fruit make contact with the picking board moving vertically downward, i.e., with zero horizontal velocity, minimizing the impact on the fruit, the contact point should be positioned slightly above the center of mass of the fruit. The installation height, H, of the picking board must satisfy the following equation.


(7) $$\text{H}=\text{L}-\text{h}+\frac{\text{R}}{\text{λ}}$$

In order to ensure the stable transfer of picked fruit to the transport mechanism, the application point of the fruit-picking plate should be slightly above the centroid of the cut crop. Furthermore, the installation height of the fruit-picking plate must satisfy the following equation:


(8) $$\text{H}\geq \frac{1}{5}\left(\text{L}-\text{h}\right)+\text{R}$$

The height range of H = 653.5 to 753.5 mm is derived by combining Equations 7, 8. Taking into account the varying growth heights of pineapple plants, the design allows for an adjustable height range of 650–720 mm.

#### 3.2.3 Fruit-picking board forward distance

Pineapple plants tend to topple during their growth, necessitating the forward displacement of the picking plate’s axis by a distance B to facilitate the collection of the fallen fruits. Additionally, the picking plate must exert a backward and downward force on the fruits post-harvest. Hence, the formula for its maximum forward displacement is as follows:


(9) $${\text{B}}_{\text{max}}=\frac{\text{R}}{\text{λ}}\sqrt{{\text{λ}}^{2}-1}$$

The front distance should satisfy the requirement of the following equation: B ≤ B_max_, After bringing the data into Equation 9 and solving for B ≤ 219 mm, 100 mm is taken here.

#### 3.2.4 Picking board speed

The rotational speed *n* of the picking plate can be determined from the established plucking speed ratio and the forward speed *vm* of the harvester, as calculated below:


(10) $$\{\begin{array}{c} v_{b}=\frac{2\pi Rn}{60}\\ \text{λ}=\frac{v_{b}}{v_{m}} \end{array}$$

Equation 10 can be solved by $n=\frac{60v_{m}\lambda }{2\pi R}$ .

Given a constant forward speed of the machine, increasing the plucking ratio requires an increase in the circumferential speed of the picking plate. However, this may result in a higher contact impulse between the picking plate and the pineapple fruit, subsequently leading to an increased damage force on the fruit. Therefore, it is crucial to control the rotational speed of the fruit-picking appropriately. In conclusion, the forward speed of the harvester is constrained to *vm*≤0.5m/s. As a result, the rotational speed of the picking plate ranges from 5 to 33 r/min.

#### 3.2.5 Picking board into the corner of the harvest

It is assumed that the plant remains perpendicular to the ground during operation. When the fruit picking plate makes contact with the fruit, in order to minimize the impact on the fruit, the horizontal component of the velocity of the picking plate should be zero. At this point, let ωt = φ_1. According to the Equation 2, we have $v_{m}-R\omega \sin\omega t_{1}=0$ , from which we can derive $\sin\omega t_{1}=\frac{v_{m}}{R\omega }=\frac{1}{\lambda }$ , and hence $\omega t=\varphi_{1}=23.48{}^{\circ}$ .

It is assumed that the apparatus operates with its axis perpendicular to the ground. When the fruit-picking plate first contacts the fruit, the horizontal component of the plate’s velocity must be zero to minimize impact on the fruit. At this point, let ωt = φ_1_. According to Equation 2, we have: *v_m_*−*Rω*sin*ωt*_1_=0. From this, we obtain $\sin\omega t_{1}=\frac{v_{m}}{R\omega }=\frac{1}{\lambda }$ , and thus $\omega t=\varphi_{1}=23.48{}^{\circ}$ .

## 4 Force analysis of plants during harvesting

As the pineapple fruit ripens, the cells in the calyx at the bottom of the fruit and in the area at the top of the stem undergo senescence, forming a separating layer at the junction where the stalk connects to the fruit. As ripening progresses, the separation force between the fruit and the stalk gradually diminishes. Upon the application of a specific horizontal force or bending moment, brittle fracture occurs near the calyx joint, leading to the detachment of the fruit from the stalk. In preliminary field pull-off experiments, it was observed that when a horizontal force was applied to the fruit, approximately 15% of the fractures occurred at the stalk-fruit junction, and most of the fracture was with the stalk. Investigating the causes of breakage at different locations along the stalk, as well as the minimum stress required for breakage, will aid the picking mechanism in performing the task more effectively. In the analysis of fruit stalk fractures, the following key assumptions are made: (1) the stalk is modeled as a cantilever beam during the picking process and is considered a linear elastic isotropic material; (2) the stalk’s cross-section remains perpendicular to the neutral plane throughout the picking process. The subsequent discussion is organized into two primary cases.

**Figure 5:**
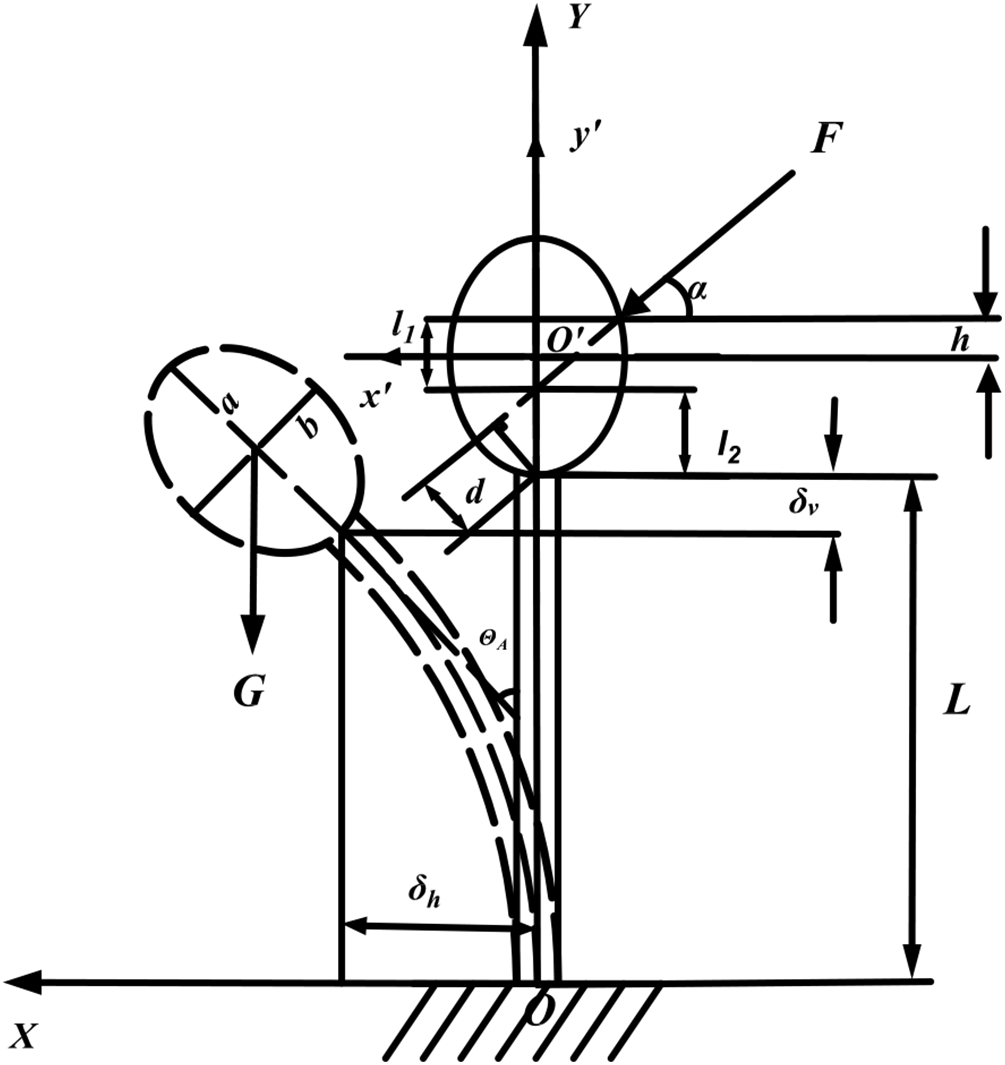
Force analysis of pineapple plant during picking.

(1) The fracture occurs at the fruit-stem union, with the corresponding force diagram presented in **Figure 5**.

In this context, *F* represents the force exerted by the picking plate on the fruit (in N), while *α* denotes the angle between this force and the horizontal plane (in °). The variable *h* refers to the vertical distance from the contact point to the central axis of the fruit (in mm). *l*_1_ is the perpendicular distance between the point where the extension of the force line intersects the *y*′-axis and the contact point (in mm). *l*_2_ represents the vertical distance between the point of intersection of the extended force line with the *y*′-axis and the end of the stem (in mm). *d* indicates the force arm of the applied force at the stem’s end (in mm). $\delta_{V}$ and $\delta_{h}$ correspond to the vertical and horizontal deformations of the stem’s end, respectively (in mm). *θ_A_* is the angle of rotation between the stem’s end and the original central axis (in °). *G* refers to the gravitational force acting on the fruit (in N); a and b are the length of the long half-axis and the short half-axis of the fruit, respectively (in mm). Lastly, *L* is the length of the stem (in mm).

Establish the right-angled coordinate systems XOY and x’O’y’, where the fixed end of the stem on the ground and the center of the pineapple fruit serve as the coordinate origins, respectively. The pineapple fruit is modeled as an ellipsoid, and the elliptic equation for the cross-section at the center of the ellipsoid is expressed as $\frac{x^{'2}}{b^{2}}+\frac{y^{'2}}{a^{2}}=1$ in the x’O’y’ coordinate system, and in the coordinate system x’O’y’:


(11) $$\{\begin{array}{c} \text{d}={\text{l}}_{2}\text{cos}\text{α}\\ {\text{l}}_{2}=\text{a}+\text{h}-{\text{l}}_{1}\\ {\text{l}}_{1}=\text{b}\sqrt{1-\frac{{\text{h}}^{2}}{{\text{a}}^{2}}}\text{tan}\text{α} \end{array}$$

Equation 11 can be solved by:


(12) $$\text{d}=\left(\text{a}+\text{h}-\text{b}\sqrt{1-\frac{{\text{h}}^{2}}{{\text{a}}^{2}}}\text{tan}\text{α}\right)$$

Let *m*=*a*+*h*, $n=b\sqrt{1-\frac{h^{2}}{a^{2}}}\tan\alpha$ , and $\eta =\frac{h}{a}$ ,. Equation 12 can then be simplified to:


(13) $$\text{d}=\sqrt{{\text{m}}^{2}+{\text{n}}^{2}}\text{sin}\left[{\text{sin}}^{-1}\left(\frac{\text{m}}{\sqrt{{\text{m}}^{2}+{\text{n}}^{2}}}\right)-\text{α}\right]$$

Here, *h* ranges from 0 to 2/3*a*, *α* spans from 0° to 23.48°, with *a*=74.9mm, *b*=56.7mm, and *d* ranges from 46.1 mm to 124.8 mm after calculating with Equation 13.

According to the impulse theorem, the following relationships hold:


(14) $$\int_{0}^{\text{t}}\text{Fdt}=\text{m}\text{v}$$

In Equation 14, *m* represents the mass of the fruit (in kg), and *v* denotes the speed after collision (in m/s).

In the XOY coordinate system, when the fruit detaches from the stem, the following equation holds for any point on the stem:


(15) $$\frac{d\text{θ}}{\text{d}\text{s}}=\frac{F\cos\text{α}\left(\text{L}-{\text{δ}}_{\text{V}}-\text{y}\right)+\left(\text{G}+\text{F}\text{sin}\text{α}\right)\left({\text{δ}}_{\text{h}}-\text{x}\right)+\text{G}\text{a}\text{sin}{\text{θ}}_{\text{A}}+\text{F}\text{d}}{\text{E}\text{I}}$$

Here, *x* and *y* represent the coordinates of a point on the stem in the XOY coordinate system, respectively, and *s* denotes the distance from the fixed end to a point along the stem.

Based on the equations $\frac{dx}{ds}=\sin\theta$ and $\frac{dy}{ds}=\cos\theta$ , the derivation of both sides with respect to *s* is given by


(16) $$\frac{{{\text{d}}^{2}}_{\text{θ}}}{{\text{d}}_{{\text{s}}^{2}}}=-\frac{\text{F}\text{cos}\text{α}\text{cos}\text{θ}+\left(\text{G}+\text{F}\text{sin}\text{α}\right)\text{sin}\text{θ}}{\text{E}\text{I}}$$

Let $\epsilon =\frac{s}{L}$ , then we obtain $\frac{d\theta }{ds}=\frac{d\theta }{Ld\epsilon }$ , and consequently, Equation 16 can be transformed as follows:


(17) $$\frac{{{\text{d}}^{2}}_{\text{θ}}}{{\text{d}}_{{\text{ϵ}}^{2}}}=-\frac{\text{F}{\text{L}}^{2}\text{cos}\text{α}\text{cos}\text{θ}+\left(\text{G}+\text{F}\text{sin}\text{α}\right){\text{L}}^{2}\text{sin}\text{θ}}{\text{E}\text{I}}$$

Let $\frac{\left(G+F\sin\alpha \right)L^{2}}{EI}=p$ and $\frac{FL^{2}\cos\alpha }{EI}=q$ , then Equation 15 can be simplified as follows:


(18) $$\frac{{\text{d}}^{2}\text{θ}}{\text{d}{\text{ϵ}}^{2}}=-\left(\text{p}\text{s}\text{i}\text{n}\text{θ}+\text{q}\text{cos}\text{θ}\right)=-\text{sin}\left(\text{θ}+\beta \right)$$

In Equation 18, $\cos\beta =\frac{p}{\sqrt{p^{2}+q^{2}}}=\frac{G+F\sin\alpha }{\sqrt{{\left(G+F\sin\alpha \right)}^{2}+{\left(F\cos\alpha \right)}^{2}}}$ and $\sin\beta =\frac{q}{\sqrt{p^{2}+q^{2}}}=\frac{F\cos\alpha }{\sqrt{{\left(G+F\sin\alpha \right)}^{2}+{\left(F\cos\alpha \right)}^{2}}}$ .

**Figure 6:**
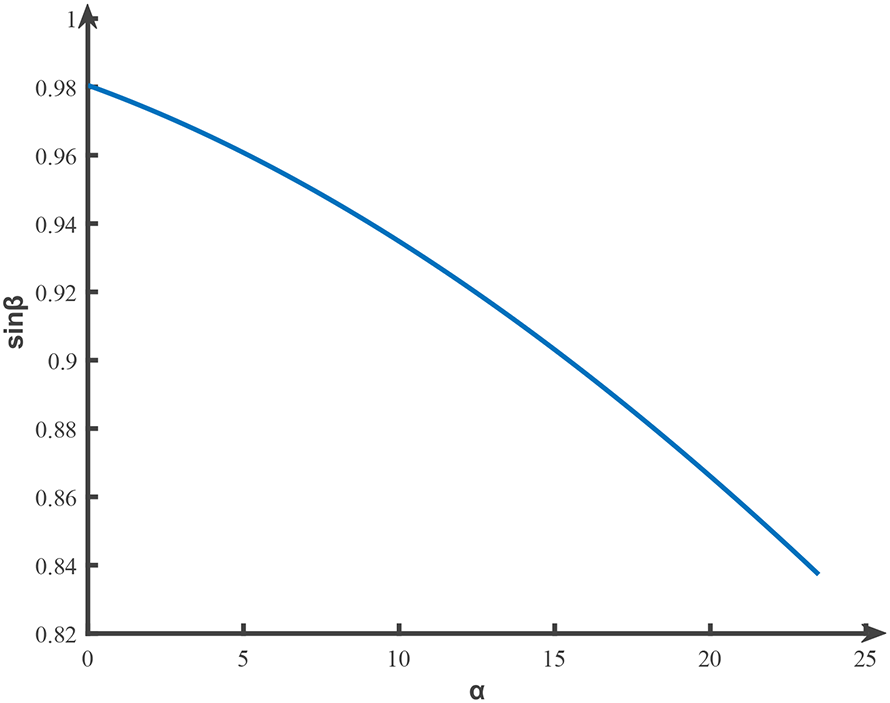
Range of values of sinβ.

Based on the previous field test, the average force required to detach the fruit and stalk horizontally was 60.45 N; thus, the value of *F* was assigned as 60.45 N. By substituting the data into Equation 17 and employing Matlab to generate the plot of sin*β*, as depicted in **Figure 6**, the value of *β* was calculated to range from 56.85° to 78.66°.

The nonlinear shooting method is employed to transform the Boundary Value Problem (BVP) into an Initial Value Problem (IVP) (Banerjee et al., 2008), assuming a curvature at the fixed end, i.e., ${\frac{d\theta }{d\epsilon }|}_{\epsilon =0}$ . The differential equations are solved using the initial conditions by the Runge-Kutta method, and the assumed initial conditions are iteratively adjusted until the error criteria are met. The corresponding Initial Value Problem (IVP) is expressed as follows:

Differential equation:


$$\frac{{\text{d}}^{2}\text{θ}}{\text{d}{\text{ϵ}}^{2}}=-\text{sin}\left(\text{θ}+\text{β}\right)$$

Initial condition:


$$\{\begin{array}{c} {\text{θ}|}_{\text{ϵ}=0}=0\\ {\frac{\text{d}\text{θ}}{\text{d}\text{ϵ}}|}_{\text{ϵ}=0}={\text{m}}_{\text{k}} \end{array}$$

The calculation procedure is outlined as follows:

1) Let *θ* be denoted as *y_1_*, and $\frac{d\theta }{d\epsilon }$ as *y_2_*. Consequently, Equation 16 simplifies to $\frac{dy_{2}}{d\epsilon }=-\sin\left(y_{1}+\alpha \right)$ ;
2) Assuming the fixed-end slope $\frac{d\theta }{d\epsilon }$ , i.e., denoted as y_2_, takes two assumed values, *m_k-1_* and *m_k_* ( k≥2 ), the differential equations in Equation 16 are solved numerically in MATLAB using the Runge-Kutta method. This is done based on the initial conditions and the assumed initial value of *y_2_*, yielding the values of *y_1_* and *y_2_*.
3) The error $\theta_{error}=\left|y_{2}-\frac{\left(Ga\sin y_{1}+Fd\right)L}{EI}\right|$ should be calculated, and the termination condition of the process is set to $\theta_{error}<10^{-6}$ .
4) The termination condition is checked; if it is not satisfied, the secant method is employed to determine a new assumed value.

$$•\;{\text{m}}_{\text{k}+1}={\text{m}}_{\text{k}}-\left[\frac{{\text{m}}_{\text{k}}-{\text{m}}_{\text{k}-1}}{{\text{θ}}_{\text{e}\text{r}\text{r}\text{o}\text{r}}({\text{m}}_{\text{k}})-{\text{θ}}_{\text{e}\text{r}\text{r}\text{o}\text{r}}({\text{m}}_{\text{k}-1})}{\text{θ}}_{\text{e}\text{r}\text{r}\text{o}\text{r}}({\text{m}}_{\text{k}})\right],(\text{k}\geq 2)$$

Here, $\theta_{error}\left(m_{k}\right)$ and $\theta_{error}\left(m_{k-1}\right)$ represent the errors corresponding to the assumed values *m_k-1_* and *m_k_*, respectively.

5) The process outlined in steps (2) through (4) is repeated until the termination condition is met.

After substituting the data, $\theta_{A}$ is calculated to range from 13.53° to 90.48°.

At the point when the fruit separates from the stem at the fruit-stem union, the moment of force at the stem end is given by: $M_{1}=Ga\sin\theta_{A}+Fd$ .

When h = 0 and α = 23.48°, M reaches its minimum value, yielding M = 2.99 N·m upon substitution of the data. Using the equation $\sigma =\frac{M}{W}=\frac{M}{\pi d^{3}/32}$ , it is found that σ = 1.71 MPa at this stage. Consulting the literature reveals that the stress at the point of separation of the fruit and stem at the calyx connection is σ = 0.94 MPa (Xing et al., 2020), indicating that the picking process can be completed at this time.

(2)When the fruit and stem separate at the thinner section of the stem below the fruit-stem union, the stem undergoes bending and deformation. According to the definition of the flexure curve, the following is observed:


(19) $$\frac{1}{\rho \left(L_{1}\right)}=\frac{M}{EI}=\frac{F\cos\alpha \left(L-\delta_{V}-y\right)+\left(G+F\sin\alpha \right)\left(\delta_{h}-x\right)+Ga\sin\theta_{A}+Fd}{EI}$$

*ρ(L_1_)* represents the radius of curvature at any point along the stem L. The radius of curvature of the flexure curve is maximized at the stem’s distal end, so at the end of the stalk Equation 19 can be reduced to Equation 20:


(20) $$\rho =\frac{EI}{Ga\sin\theta_{A}+Fd}$$

In the region near the calyx, the pineapple stalks exhibit minimal variation in diameter. These can be regarded as uniformly varying cylinders, where the neutral layer exhibits the same curvature as the compressive and tensile layers. The corresponding lengths of the layers within the range of dθ are as follows:


(21) $$\{\begin{array}{c} ds_{1}=\rho d\theta \\ ds_{c}=\left(\rho -\frac{1}{2}d_{s}\right)d\theta \\ ds_{t}=\left(\rho +\frac{1}{2}d_{s}\right)d\theta \end{array}$$

Let *d_s_* denote the diameter of the stem end, $ds_{1}$ represent the length of the neutral layer, $ds_{c}$ is the length of the compressive layer, and $ds_{t}$ is the length of the tensile layer. The tensile stress associated with the deformation within the specified range is as follows:


(22) $$\{\begin{array}{c} d\sigma_{c}=Eds_{c}\\ d\sigma_{t}=Eds_{t} \end{array}$$

Combining Equations 21, 22, the stem is subjected to the maximum tensile and compressive stresses at *L*_1_=*L* as shown in Equation 23.


(23) $$\{\begin{array}{c} \sigma_{cmax}=\int_{0}^{\rho +\frac{1}{2}d_{s}}d\sigma_{c}\\ \sigma_{tmax}=\int_{0}^{\rho -\frac{1}{2}d_{s}}d\sigma_{t} \end{array}$$

$\sigma_{cmax}$ denotes the maximum compressive stress experienced by the stem at *L*_1_=*L*, and $\sigma_{tmax}$ represents the maximum tensile stress at *L*_1_=*L*.

When the pineapple is pushed by the fruit picking board, the parameters F1, h and θ can be considered as constant for any point on the stem. As a result, the compressive and tensile stresses at any point on the stalk are solely dependent on the height *L*_1_ from the bottom of the stalk. Based on the previous analysis, it is evident that the deformation at *L*_1_=*L* is maximal, and the corresponding stress is also at its maximum (Liu et al., 2023). When *L*_1_ exceeds a certain threshold, the tensile and compressive stresses at this point on the stalk become greater than the maximum shear stress the stalk can withstand when the pineapple is not fully ripe, but less than the minimum shear stress required for fracture at the calyx, denoted as *L_T_*. Consequently, when the pineapple is subjected to the action of the fruit-picking plate, fracture may occur at *L_T_* ∼*L*.

## 5 Rigid-flexible coupling simulation verification

### 5.1 Simulation modeling

This study employs and analyzes a rigid-flexible coupling simulation of the fruit-picking plate and the pineapple plant using ADAMS software. A dynamic simulation model of the picking mechanism and the pineapple plant is developed, and a flexible body model of the pineapple plant is constructed within ADAMS.

#### 5.1.1 Modeling of flexible bodies in pineapple plants

A 3D model of the pineapple plant was constructed using pre-measured plant parameters and the 3D modeling software SolidWorks. Due to limitations in computer simulation performance, the pineapple plant was simplified by removing the leaves and crown buds, retaining only the stalks and fruits. The 3D model of the pineapple plant stem was imported into ADAMS (I. et al., 2020; Xing et al., 2020), and the mechanical properties of the fruit and stalk were defined according to the data in **Tables 1**, **2**: the fruit density was 874 kg/m³, Young’s modulus was 2.67 MPa, Poisson’s ratio was 0.297; the stalk density was 1130 kg/m³, Young’s modulus was 24.28 MPa, and Poisson’s ratio was 0.44. Subsequently, ANSYS APDL software is employed to construct the flexible body of the stem and export the MNF file, which is then imported into ADAMS.

**Table 1:** Measurements of biological characteristics of pineapple plants.

Average fruit mass/g	Fruit mean long-axis length/mm	Fruit mean short-axis length/mm	Fruit density/*(*kg/m^3^ *)*	Average stem diameter/mm	Average stem length/mm	Stem density/*(*kg/m^3^ *)*
1237.27	149.8	113.4	874	26.11	499.8	1130

**Table 2:** Measurements of plant mechanical properties.

Fruit pulp modulus of elasticity/MPa	Fruit pulp Poisson’s ratio	Modulus of elasticity of stem/MPa	Stem Poisson’s ratio	The horizontal tensile breaking force of fruit stem union/N
2.67	0.297	24.28	0.44	60.45

#### 5.1.2 Coupled modeling

A three-dimensional model of the cutting table is established, simplified, and imported into ADAMS software. The Boolean operation is employed to merge the connecting arm with its fixed component into a unified structure. Additionally, a rotating vice is placed between the rotary axis and the frame, a fixed vice between the connecting arm and the rotary axis, and a sliding vice between the frame and the ground. Rotary and linear drives are incorporated into the rotating and sliding components. ADAMS does not directly apply flexible links to flexible bodies, such as bushings, beams, or field elements. In order to utilize these flexible links, a dummy object must be attached to the flexible body, wherein the mass and moment of inertia of the object are set to zero, and only its geometry is preserved. To achieve this, the rigid body of the stem is modeled as a dummy object, which is then connected to the flexible body of the stem using a fixing vice, followed by the addition of a flexible coupling to the dummy object (Tao et al., 2012). Bushings are placed between the root of the stalk and the ground, as well as between the fruit and the stalk, with a contact stiffness of 600 N/mm and a damping coefficient of 600 N/(mm/s). Contact is established between the picking plate and the fruit, modeled as a rigid body, with a contact stiffness of 140 N/mm, a force index of 2.2, a damping coefficient of 0.5 N/(mm/s), and a contact depth of 0.01 mm. Friction is introduced between the harvesting mechanism and the fruit, defined according to Coulomb’s law. The default setting parameters are used, with static and dynamic friction coefficients between the fruit and the picking plate set at 0.3 and 0.1, respectively.

**Figure 7:**
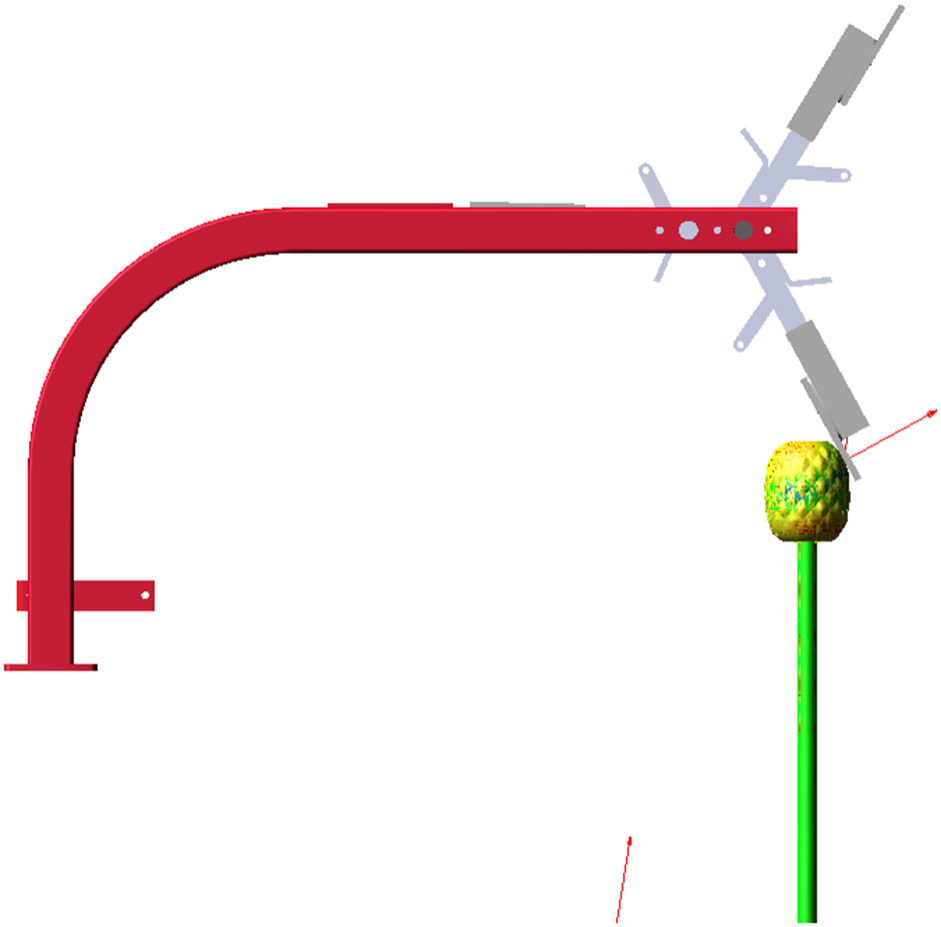
Adams simulation model.

**Figure 8:**
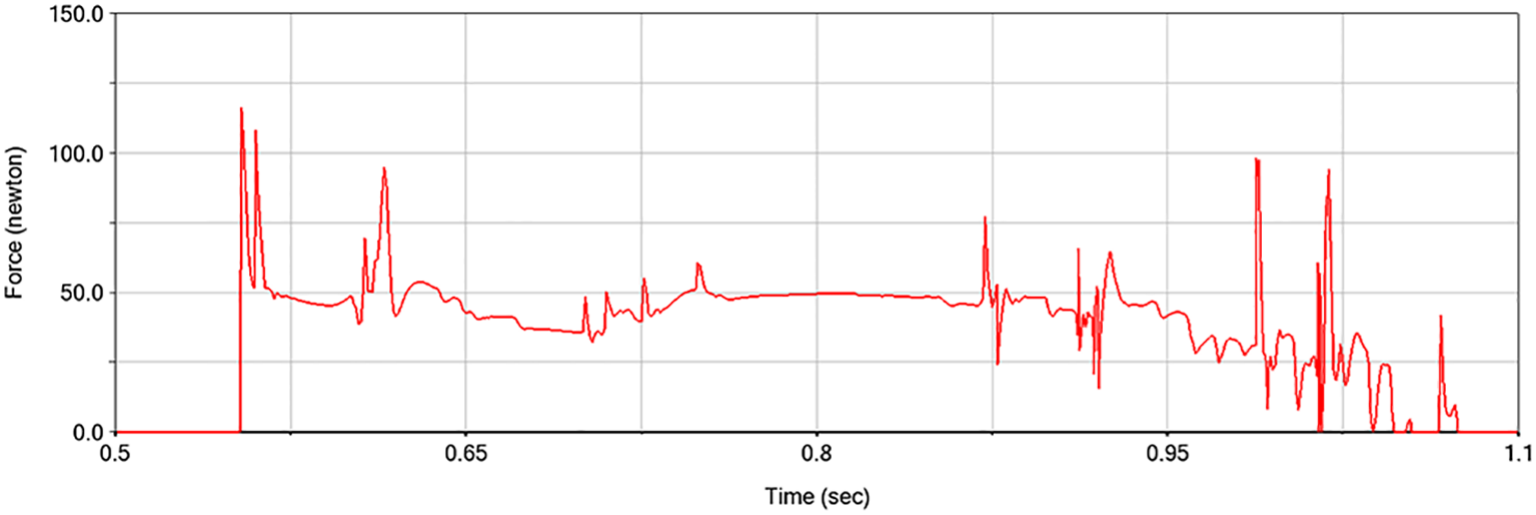
Fruit picking plate and fruit force during the picking process.

Simulation scripts are employed to reduce the sleeve force between the stem and the fruit, based on the moment imparted to the fruit by the picking plate, in order to simulate the fruit-picking process. The simulation step size was set to 0.001 seconds, and the total simulation duration was adjusted in accordance with the harvesting speed. The simulation model is presented in **Figure 7**. The force between the pineapple fruit and the fruit-picking plate can be retrieved from the post-processing interface following the simulation, as shown in **Figure 8**.

### 5.2 Simulation experimental methods

The test factors were identified as the paddle speed ratio, cutting table height, and forward speed (Gao et al., 2020; Huang et al., 2020; Li et al., 2024). The test indices were defined as the effective value of the force between the fruit-picking plate and the pineapple fruit (denoted as Y_1_), and the maximum force (denoted as Y_2_). The effective value of the force is represented by the root-mean-square (RMS) value of the interaction force during contact between the fruit and the fruit-picking plate. A three-factor, five-level central composite design (CCD) was employed to model the functional relationship between the test factors and the response variables, utilizing multivariate quartic equations to determine the optimal parameter combinations under varying conditions.

In conjunction with the parameters of the pineapple harvester’s cutting table, the theoretically derived values for the plucking speed ratio, forward speed, and the height of the fruit-picking plate were utilized as the parameter ranges for the multifactor combination test. Based on the theoretical calculations, the plucking speed ratio ranges from 1.6 to 2.0; the forward speed ranges from 0.1 to 0.5 m/s; the height of the fruit-picking plate ranges from 860 to 960 mm. Additionally, the practical force exerted between the fruit-picking plate and the fruit should exceed the average pulling force. In contrast, the maximum force should be minimized as a reference, and the coding of the experimental factors is presented in **Table 3**.

**Table 3:** Experimental factor coding.

Code	Factor		
	Pulling Speed Ratio A	Picking Board Height B/mm	Forward Speed C/(m/s)
-2	1.6	860	0.1
-1	1.7	885	0.2
0	1.8	910	0.3
1	1.9	935	0.4
2	2.0	960	0.5

### 5.3 Experimental results and analysis

The simulation experiment design and its corresponding results are presented in **Table 4**.

**Table 4:** Experimental design scheme and results.

Experiment Number	Factor			Y_1_/N	Y_2_/N
	A	B/mm	C/(m/s)		
1	1.7	885	0.2	31.3395	142.9357
2	1.9	885	0.2	40.4133	191.3943
3	1.7	935	0.2	27.6017	94.3919
4	1.9	935	0.2	34.9016	129.0902
5	1.7	885	0.4	52.3018	248.2687
6	1.9	885	0.4	71.5534	356.0237
7	1.7	935	0.4	47.6086	199.0955
8	1.9	935	0.4	66.1754	266.6405
9	1.6	910	0.3	34.9121	99.1618
10	2	910	0.3	56.4382	186.6458
11	1.8	860	0.3	50.0201	150.0095
12	1.8	960	0.3	49.4198	237.4268
13	1.8	910	0.1	23.5089	81.8356
14	1.8	910	0.5	73.0233	254.3006
15	1.8	910	0.3	45.6294	159.2487
16	1.8	910	0.3	45.6467	175.2458
17	1.8	910	0.3	45.6872	116.1517
18	1.8	910	0.3	45.2883	117.5823
19	1.8	910	0.3	45.6185	116.9289

The collision forces were analyzed using ANOVA with Design-Export software, and insignificant terms were excluded. The results are presented in **Table 5**.

**Table 5:** Analysis of variance.

Test indicators	Source of variance	Sum of squares	Degrees of freedom	F	P
Y_1_	Model	3297.46	10	4956.08	< 0.0001
	A	231.69	1	3482.25	< 0.0001
	C	1225.84	1	18424.34	< 0.0001
	AB	0.76	1	11.36	0.0098
	AC	57.48	1	863.99	< 0.0001
	B^2^	26.37	1	396.29	< 0.0001
	C^2^	11.03	1	165.82	< 0.0001
	A^2^B	46.66	1	701.33	< 0.0001
	A^2^C	1.18	1	17.81	0.0029
	AB^2^	7.76	1	116.57	< 0.0001
	A^2^B^2^	2.95	1	44.29	0.0002
	Residual	0.53	8		
	Lack of Fit	0.43	4	4.08	0.1010
	Pure Error	0.10	4		
	R^2^	0.9998			
	Cor Total	3297.99	18		
	Model	84419.66	6	20.21	< 0.0001
Y_2_	A	11741.07	1	16.86	0.0015
	B	3820.89	1	5.49	0.0372
	C	45918.74	1	65.94	< 0.0001
	B^2^	3846.69	1	5.52	0.0367
	A^2^B	11248.67	1	16.15	0.0017
	A^2^B^2^	9725.93	1	13.97	0.0028
	Residual	8355.88	12		
	Lack of fit	5183.55	8	647.94	0.6270
	Pure Error	3172.33	4	793.08	
	R^2^	0.9099			
	Cor Total	92775.54	18		

As shown in **Table 5**, the P-value for the effective force variance model is less than 0.0001, indicating that the regression model is highly significant. The regression equation for the effective force between the picking plate and the fruit is as Equation 24:


(24) $$Y_{1}=45.6+5.38\lambda +12.38v_{m}-0.31\lambda H+2.68\lambda v_{m}+1.03H^{2}+0.67v_{m}-2.42\lambda^{2}H+0.54\lambda^{2}v_{m}+1.39\lambda H^{2}-0.81\lambda^{2}H^{2}$$

**Figure 9:**
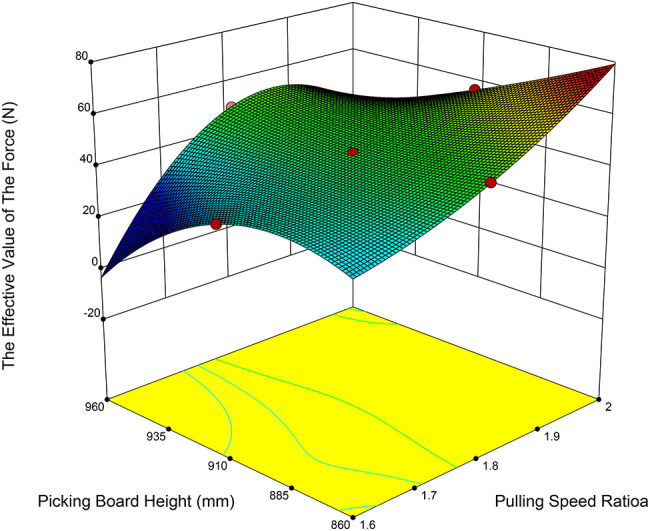
Response surface of plucking speed ratio and height of picking plate to the RMS value of force.

**Figure 10:**
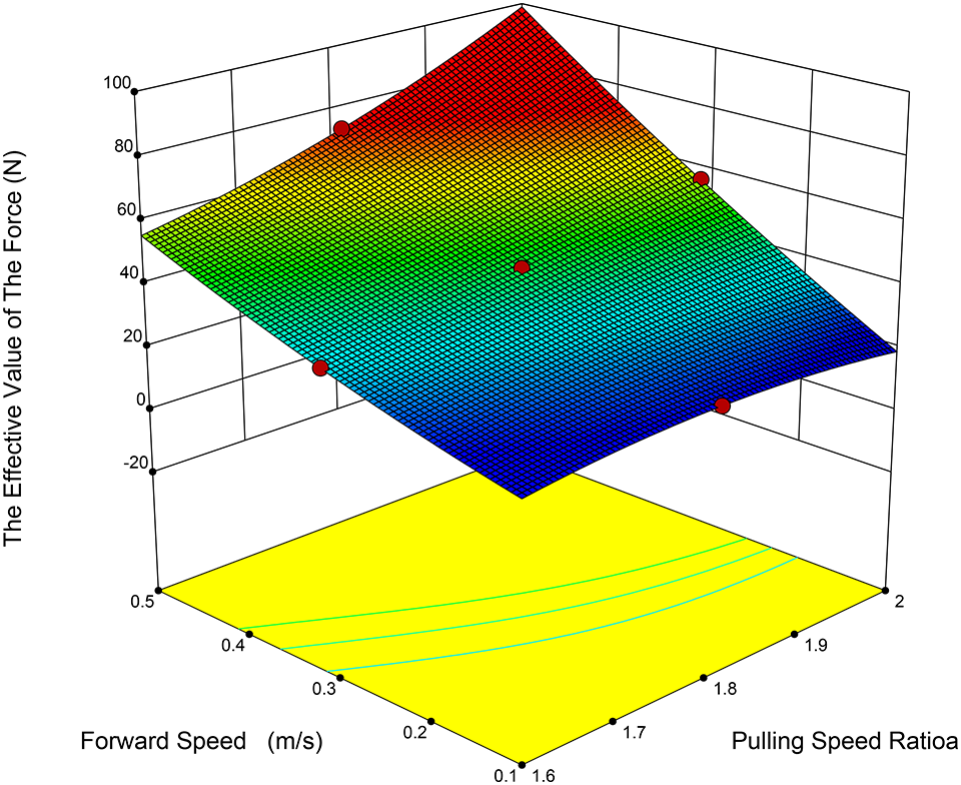
Response surface of plucking speed ratio and forward speed to the RMS value of force.

Based on the analysis of the test results, the two most influential factors on the effective value of the force were identified as the plucking speed ratio (A) and forward speed (C), with the influence ranking as follows: forward speed, followed by plucking speed ratio. The interactions were analyzed, as depicted in **Figures 9**, **10**, revealing significant interactions between the plucking speed ratio and the height of the picking board, as well as between the plucking speed ratio and the forward speed. When the plucking speed ratio ranges from 1.6 to 1.7, the effective value of the force first increases and then decreases with the height of the fruit-picking board. For a plucking speed ratio of 1.7 to 1.9, the effective force value remains relatively unchanged as the height of the fruit-picking board increases. In contrast, when the plucking speed ratio falls between 1.9 and 2.0, the effective force value decreases as the height of the fruit-picking board rises. When the height of the fruit-picking plate is between 860 and 925 mm, the effective value of the collision force increases with the plucking speed ratio. In contrast, when the height ranges from 925 to 960 mm, the collision force first increases and then decreases as the plucking speed ratio increases. When the plucking speed ratio remains constant, the effective value of the force is positively correlated with forward speed. Conversely, when the forward speed remains constant, the effective force value is positively correlated with the plucking speed ratio. The primary reason for this is that the plucking speed ratio and forward speed determine the relative speed of the fruit-picking plate. As the plucking speed ratio and forward speed increase, the speed of the fruit-picking board relative to the direction of pineapple harvesting also increases, which in turn leads to an increase in the action force exerted on the pineapple.

As shown in **Table 5**, the P-value for the variance model of the maximum collision force is below 0.0001, indicating a highly significant regression model. Consequently, the regression equation for the maximum force between the picking plate and the fruit is derived as Equation 25:


(25) $$Y_{2}=145.23+27.09\lambda +21.85H+53.57v_{m}+66.06v_{m}+12.12H^{2}-53.03\lambda^{2}H+46.13\lambda^{2}H^{2}$$

Based on the analysis of the test results, the three factors that have the greatest influence on the maximum force were identified as the pulling speed ratio (A), the height of the picking board (B), and the forward speed (C). The order of their influence is as follows: forward speed, pulling speed ratio, and picking board height.

## 6 Parameter optimization and field experiments

### 6.1 Parameter optimization

To optimize the performance of the pineapple harvester cutting table, the effective force exerted by the picking plate on the fruit during harvesting must exceed 60.45 N. Simultaneously, to minimize fruit loss, the maximum force applied by the picking plate should be kept to a minimum. Based on the aforementioned requirements, parameter optimization is conducted to identify the optimal combination of parameters, with the optimization objective function defined as Equation 26:


(26) $$\{\begin{array}{c} minY_{2}\\ s.t.\{\begin{array}{c} \begin{array}{c} Y_{1}\geq 60.45\\ 1.6\leq A\leq 2.0 \end{array}\\ \begin{array}{c} 860\leq B\leq 960\\ 0.1\leq C\leq 0.5 \end{array} \end{array} \end{array}$$

The optimization process was conducted using Design-Export 10 software. When the plucking speed ratio is set at 1.812, the height of the fruit picking plate is 888.69 mm, and the forward speed is 0.399 m/s, the effective force is 60.45 N, and the maximum force is 192.987 N. The optimal parameter combination for the regression model was rounded to the following values: plucking speed ratio of 1.8, fruit picking plate height of 890 mm, and forward speed of 0.4 m/s. The optimal parameter combination was derived using Design-Export 10 software.

### 6.2 Field experiment

**Figure 11:**
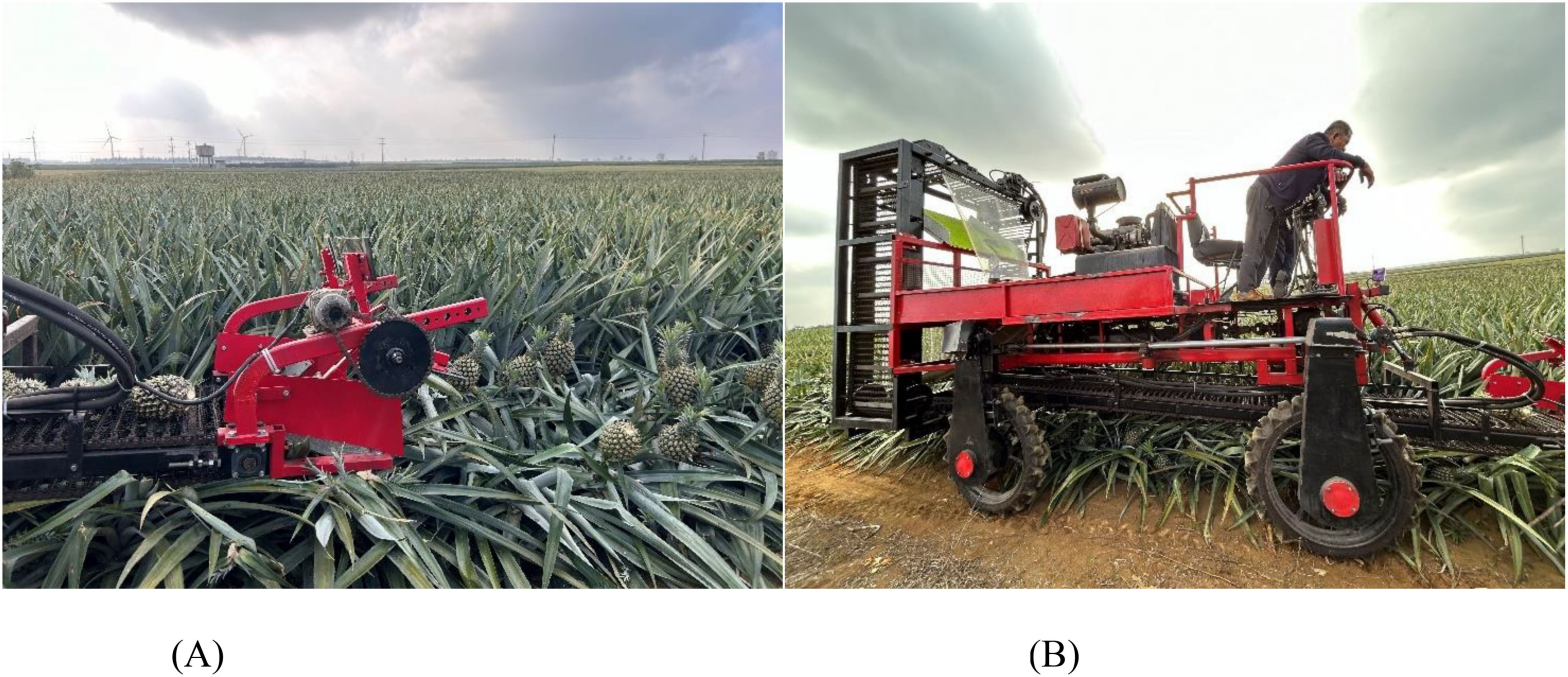
Pineapple harvester field experiment map. Figure **(A)** Cutting table for fruit picking Figure **(B)** Fruit picking by picker.

The experiment was conducted on November 20, 2024, at the pineapple planting test base of the Nuoxiangyuan Agricultural Products Cooperative in Qujie Town, Xuwen County, Guangdong Province, China. The test material was Bali species pineapple, and the experimental area was approximately 667 m². The plantation utilized a wide and narrow row configuration with a border width of approximately 1 meter and furrow width of around 40 cm. Pineapples were planted within the border at a spacing of 40 cm per row, resulting in a planting density ranging from 4,000 to 4,400 plants per 667 m². The average height of the pineapple plants was 593.7 mm, the average fruit mass was 1.29 kg, the average transverse and longitudinal fruit diameters were 189.5 mm and 226.4 mm, respectively, and the average stem diameter was 34 mm. The test equipment used was a self-propelled pineapple harvester. The pineapple harvester utilized the optimal parameter combination for harvesting operations. The harvesting tests were conducted randomly in the pineapple orchard, encompassing both ripe and unripe pineapples. The field test results are shown in **Figure 11**.

The performance of the harvesting mechanism was assessed by considering both the harvesting rate (N) and the damage rate (S). Both the harvesting rate (N) and the damage rate (S) were utilized to assess the performance of the harvesting mechanism. Since the primary damage to pineapple fruit affects the flesh, while the fruit’s waxy skin complicates visual detection of such damage, the harvested pineapples were stored at a constant temperature of 20°C for 3 days. Subsequently, the fruit was cut at the potentially damaged areas to observe discoloration of the flesh. The undamaged flesh exhibited a color nearly identical to that of the harvested fruit, whereas the damaged flesh displayed a yellowish-brown hue. Pineapple flesh discoloration was classified as undamaged if the discoloration area measured less than 10 mm in diameter. The formulas for calculating both the harvesting rate and the damage rate are presented as follows:


$$\{\begin{array}{c} N=\frac{N_{1}}{N_{1}+N_{2}}\times 100\%\\ S=\frac{S_{2}}{S_{1}+S_{2}}\times 100\% \end{array}$$

In this context, N_1_ represents the number of successfully harvested pineapples, N_2_ denotes the number of unsuccessfully harvested pineapples, S_1_ indicates the number of successfully harvested undamaged pineapples, and S_2_ refers to the number of successfully harvested damaged pineapples.

Multiple harvests were tested in several randomly selected areas where pineapples were at or near maturity. The experimental program was designed based on the simulation optimization results, combined with the actual parameters, and the results are presented in **Table 6**. Test 14 corresponds to the optimal solution of the simulation model for this group.

**Table 6:** Experimental design scheme and results.

Experiment Number	Pulling Speed Ratio	Picking Board Height/mm	Forward Speed/(m/s)	Recovery Ratio/%	Damage Rate/%
1	1.6	860	0.3	68.6	11.5
2	2.0	860	0.3	70.0	15.2
3	1.6	960	0.3	68.0	11.8
4	2.0	960	0.3	72.1	16.3
5	1.6	910	0.1	70.4	14.1
6	2.0	910	0.1	70.3	13.7
7	1.6	910	0.5	70.6	14.2
8	2.0	910	0.5	70.8	15.3
9	1.8	860	0.1	70.9	14.2
10	1.8	960	0.1	68.7	14.4
11	1.8	860	0.5	71.1	13.9
12	1.8	960	0.5	67.5	13.5
13	1.8	910	0.3	69.6	14.0
14	2.0	910	0.3	71.3	14.8

By utilizing an optimized combination of parameters—specifically, a plucking speed ratio of 1.8, a plucking wheel height of 890 mm, and a forward speed of 0.4 m/s—a superior harvesting effect can be achieved. Under these conditions, the harvesting rate is 71.3%, and the damage rate is 14.8%.

In this study, the harvesting device was capable of harvesting one acre of pineapples in 1.5 hours. Based on a yield of 4000–4400 pineapples per acre, the time required for harvesting a single pineapple was 1.22–1.35 seconds. The actual harvesting capacity of the assisted pineapple harvesting device developed by Kahandage et al. was 385 fruits per hour (Kahandage et al., 2021). The semi-automatic pineapple harvester developed by Singh et al. can harvest 200 pineapples per hour (Singh et al., 2022), while the pineapple harvesting robot developed by Bhat et al. requires 21 to 24 seconds per pineapple (Bhat and Chayalakshmi, 2021). The automatic straddle pineapple harvester designed by Guo et al. achieves a harvesting efficiency of 1636 fruits per hour (Guo et al., 2021). Additionally, Liu et al. proposed a multi-flexible finger drum harvesting device, which allows for a single pineapple picking time of 1 second (Liu et al., 2022). However, this solution requires more uniform pineapple heights. Wang et al. designed an end-effector with a double V-shaped finger structure, achieving an average picking time of 23 seconds per pineapple (Wang et al., 2012). Du et al. designed an end-effector with an average picking time of 14.9 seconds per pineapple (Du et al., 2019). In contrast, the pineapple-picking robot developed by Nguyen P. T. Anh and colleagues achieves an average picking efficiency of 12 seconds per fruit (Anh et al., 2020). The solutions proposed in this paper are significantly faster than those of existing pineapple harvesting robots and hand-held harvesters, and they involve lower fabrication complexity.

In the harvesting experiments, most of the successfully harvested pineapples were separated from the plant by the action of the fruit-picking plate, resulting from the breakage of the stem below the abscission layer at the calyx. A small portion of the abscission layer at the calyx was also separated from the plant. The reasons for unsuccessful harvests were primarily associated with plant inclination angles, as well as leaf and plant density. Some pineapple plants were not positioned vertically relative to the ground, and in some cases exhibited significant tilt due to various factors during growth. As a result, the fruit did not make proper contact with the picking plate, or in certain instances, it was below the operational range of the plate, causing insufficient stress on the stem, preventing it from breaking or the fruit from detaching. The plant leaves were taller and denser, which, combined with the tilted fruit, led to the picking plate initially contacting the leaves. This caused the entire plant to bend, preventing the fruit and stem from experiencing sufficient force during collision. The plant density was excessive, and the dense leaves caused a squeeze, resulting in some fruits being too closely spaced and thus missing the harvest. The main cause of harvesting damage is the tilting of the stem, oblique contact between the picking board and the fruit, which prevents the fruit from being peeled in a single contact, leading to continuous collision damage. Another contributing factor to the damage is the fruit falling from a height during the collection process. These findings suggest that utilizing a rotating fruit picker plate to apply a breaking moment to the pineapple can effectively facilitate harvesting. The outcomes of this study provide valuable insights for the development of pineapple harvesting mechanisms and offer theoretical guidance for mechanized harvesting practices.

## 7 Conclusions

Based on the physical and mechanical properties of pineapple, a self-propelled pineapple harvester cutting table structure was designed to mimic hand-picking methods. The key components of the cutting table were analyzed and optimized, using the following parameters: the radius of the picking plate is 280 mm, the height of the picking plate from the ground is 890 mm, the plucking speed ratio is 1.8, and the forward speed is 0.4 m/s. Based on the principle of large deflection and the assumption of a cantilever beam, a mechanical model of the separation process between the pineapple fruit and stem was analyzed and developed. This model establishes the mechanical basis for pineapple fruit-stem separation and offers valuable insights for future research in pineapple harvesting. A simulation model was developed to study the rigid-flexible coupling between the fruit picker and the pineapple plant, followed by a multi-factor optimization experiment. Mathematical models were developed to relate the effective force exerted by the fruit-picking board on the pineapple fruit to the pulling speed ratio and forward speed, as well as the maximum force to the pulling speed ratio, the height of the fruit-picking board, and the forward speed. The optimal operating parameters were determined to be a pulling speed ratio of 1.8, a picking plate height of 890 mm, and a forward speed of 0.4 m/s. Field experiments demonstrated that the pineapple harvester cutter functioned effectively, successfully separating the pineapple fruit from the plant. Under the optimal parameters, the harvesting rate was 71.3%, and the damage rate was 14.8%.

## Data Availability

The original contributions presented in the study are included in the article/supplementary material. Further inquiries can be directed to the corresponding author.
